# Genetic Regulation of Liver Metabolites and Transcripts Linking to Biochemical-Clinical Parameters

**DOI:** 10.3389/fgene.2019.00348

**Published:** 2019-04-17

**Authors:** Siriluck Ponsuksili, Nares Trakooljul, Frieder Hadlich, Karen Methling, Michael Lalk, Eduard Murani, Klaus Wimmers

**Affiliations:** ^1^Leibniz Institute for Farm Animal Biology (FBN), Institute for Genome Biology, Functional Genome Analysis Research Unit, Dummerstorf, Germany; ^2^Institute for Biochemistry – Metabolomics, University of Greifswald, Greifswald, Germany; ^3^Faculty of Agricultural and Environmental Sciences, University of Rostock, Rostock, Germany

**Keywords:** mQTL, eQTL, metabolite, transcript, SNPs, biochemical-clinical traits, biomarker, pig model

## Abstract

Given the central metabolic role of the liver, hepatic metabolites and transcripts reflect the organismal physiological state. Biochemical-clinical plasma biomarkers, hepatic metabolites, transcripts, and single nucleotide polymorphism (SNP) genotypes of some 300 pigs were integrated by weighted correlation networks and genome-wide association analyses. Network-based approaches of transcriptomic and metabolomics data revealed linked of transcripts and metabolites of the pentose phosphate pathway (PPP). This finding was evidenced by using a NADP/NADPH assay and *HDAC4* and *G6PD* transcript quantification with the latter coding for first limiting enzyme of this pathway and by RNAi knockdown experiments of *HDAC4*. Other transcripts including *ARG2* and *SLC22A7* showed link to amino acids and biomarkers. The amino acid metabolites were linked with transcripts of immune or acute phase response signaling, whereas the carbohydrate metabolites were highly enrich in cholesterol biosynthesis transcripts. Genome-wide association analyses revealed 180 metabolic quantitative trait loci (mQTL) (*p* < 10^-4^). Trans-4-hydroxy-L-proline (*p* = 6 × 10^-9^), being strongly correlated with plasma creatinine (CREA), showed strongest association with SNPs on chromosome 6 that had pleiotropic effects on *PRODH2* expression as revealed by multivariate analysis. Consideration of shared marker association with biomarkers, metabolites, and transcripts revealed 144 SNPs associated with 44 metabolites and 69 transcripts that are correlated with each other, representing 176 mQTL and expression quantitative trait loci (eQTL). This is the first work to report genetic variants associated with liver metabolite and transcript levels as well as blood biochemical-clinical parameters in a healthy porcine model. The identified associations provide links between variation at the genome, transcriptome, and metabolome level molecules with clinically relevant phenotypes. This approach has the potential to detect novel biomarkers displaying individual variation and promoting predictive biology in medicine and animal breeding.

## Introduction

Metabolites are substrates or products of metabolism. As one of the main “omics-” technologies, metabolomics can bridge the phenotype–genotype gap due to the close association of metabolites to cellular biochemical processes (Cascante and Marin, 2008). The metabolome represents the final “omics-” level in the genotype–phenotype map and reflects changes in phenotype and function, whereas the transcriptome and proteome act as mediators of flow (Ryan and Robards, 2006). High-performance metabolic profiling is a high-throughput analysis suitable for routine measurement of endogenous metabolites and metabolic signatures related to health issues (Johnson et al., 2010). Recent advances in bio-analytical technologies allow genome-wide association studies with metabolomics (mGWAS) based on the assumption that the biochemical function of a gene variant is reflected by varied metabolite levels, which are substrates, products, or ligands of that gene product (Adamski and Suhre, 2013).

Association of a single nucleotide polymorphism (SNP) with a metabolic trait indicates that the metabolic phenotype is either a cause or consequence of the metabolic state. Accordingly, it allows generation of biological hypotheses about the role of that metabolite for organismal phenotype (Kathiresan et al., 2009; Franke et al., 2010). Several studies have reported metabolic quantitative trait loci (mQTL) or mGWAS for serum metabolite concentrations in humans (Gieger et al., 2008; Illig et al., 2010; Nicholson et al., 2011). Genetic influences on blood metabolites in healthy humans can be detected by combining genetic variants and metabolic traits (Shin et al., 2014; Draisma et al., 2015).

The regulatory mechanisms between transcript and metabolite levels are still not well understood. Thus, integrating transcriptomics and metabolomics can elucidate the relationship between genes and their transcripts, metabolites, and outcome levels in cells, as reported in microbial, plant, and animal systems (Hoefgen and Nikiforova, 2008; Yang et al., 2009; Yabushita et al., 2013). Expression quantitative trait loci (eQTL) studies are a powerful functional genomics tool, revealing genetic loci that affect RNA transcription levels. eQTL studies facilitate uncovering biological mechanisms that mediate gene regulation and building complex molecular networks for metabolic, biochemical-clinical, and hematological traits (Ponsuksili et al., 2011, 2012, 2016). eQTL studies suggest the potential value of complementary association studies with other molecular traits, such as endocrine or metabolic phenotypes (Ponsuksili et al., 2012; Ghazalpour et al., 2014).

Given the central role of the liver in metabolic and immune functions, we hypothesized that variation of traits related to metabolic state and performance are largely reflected by metabolites and transcripts of hepatic metabolic pathways. Herein, we characterized the genetic landscape of porcine liver metabolites and we linked hepatic metabolite profiles and transcriptomes as well as plasma biochemical-clinical traits in pigs. Analyses of trait-correlated hepatic metabolites and mQTL, together with our previous eQTL results, provide a fine map of loci controlling metabolic profiles. Because pigs are valuable models, this knowledge provides a rational basis not only for understanding pig physiology, but also for human medical research.

## Materials and Methods

### Animals and Sample Collection

Pigs from a German Landrace herd were reared, performance tested, sampled, and used for genome-wide association studies of liver metabolites. Animal care and tissue collection procedures were approved by the Animal Care Committee of the Leibniz Institute for Farm Animal Biology and carried out in accordance with the approved guidelines for safeguarding good scientific practice at the institutions of the Leibniz Association. Measures have been taken to minimize pain and discomfort in line with the guidelines laid down in the Council Directive 86/609/EEC of 24 November 1986. Veterinary inspection of live pigs and their carcasses and organs after slaughter confirmed a lack of any impairments, disease symptoms, or pathological signs to avoid any bias of blood phenotypes. Liver and blood samples were collected from pigs at an average age of 170 days at the experimental slaughter facility of the Leibniz Institute for Farm Animal Biology, between 8.00 and 10.00 in the morning.

### Plasma Analyte Measurement

Plasma cortisol concentrations (total) were determined using commercially available enzyme-linked immunosorbent assays (DRG, Marburg, Germany), performed in duplicate according to the manufacturer’s protocol. Biochemical-clinical parameters of blood samples were determined using an automated analyser device (Fuji DriChem 4000i, FujiFilm, Minato, Japan) including albumin (ALB), ammonia nitrogen (NH_3_), blood urea nitrogen (BUN), glucose (GLU), inorganic phosphorus (IP), and creatinine (CREA).

### Metabolic Profiling

A total of 350 individual porcine livers from the same animals used for biochemical-clinical blood plasma analyses were subjected to metabolite profiling. Liver was ground under liquid nitrogen into a homogeneous mixture before being divided for extraction using two-step extraction methods from Wu et al. (2008). We homogenized 50 mg frozen liver powder in 4 mL/g cold methanol and 0.85 mL/g cold water in homogenization tubes containing ceramic beads. Three internal standards were used, including 1 mM ribitol and 0.2 mM palmitic acid-d31 for GC-MS, 250 μM camphorsulphonic acid for LC-MS. Homogenates were transferred to 1.8-mL glass vials and mixed with 2 mL/g chloroform. Samples were vortexed for 60 s, left on ice for 10 min to partition, and centrifuged. Polar and non-polar layers were removed and dried, although we only concentrated on polar phase metabolites in this study. We analyzed samples using non-targeted metabolic profiling instrumentation combining two platforms, GC-MS and HPLC-MS. Both methods represent relative metabolite amount per liver sample (25 mg wet weight of liver per sample). After extraction, samples were split for GC-MS and HPLC-MS analysis, frozen, and lyophilized. Details of GC-MS and HPLC-MS setups are done according to manufacturer’s instructions. In brief, lyophilized samples were derivatized and centrifuged. The supernatant was transferred to a new vial before injection for GC-MS. Qualitative and quantitative analyses were performed using ChromaTOF software v4.50.8.0 (LECO Corporation, United States). HPLC-MS analysis was performed using an Agilent 1100 series liquid chromatographic system (micrOTOF, Bruker Daltonik GmbH, Germany). For analysis, lyophilized liver extracts and blank samples were dissolved in 100 μL water and centrifuged. For chromatographic separation, 5 μL of each sample were injected into a Synergi 2.5 μm Fusion RP column attached to a guard column of the same material (Supplementary Methods, Data Sheet 1). Metabolite identification was verified and analysis using the software DataAnalysis v4.0 and QuantAnalysis v2.0 (Bruker Daltonik GmbH, Germany).

### SNP Genotype and mRNA Expression Profile Data

Single nucleotide polymorphism genotyping and mRNA hepatic expression profiling was performed using samples of identical animals as for biochemical-clinical blood plasma analyses and liver metabolite profiling. In brief, genotyping was performed using the PorcineSNP60 BeadChip (Illumina Inc., San Diego, CA, United States) per the manufacturer’s SNP Infinium HD assay protocol. Samples with call rates of <99%, markers with low minor-allele frequency (<5%), and markers that strongly deviated from Hardy–Weinberg equilibrium (*p* < 0.0001) were excluded. The average call rate for all samples was 99.8% ± 0.2 after filtering.

Total RNA was isolated from liver and amplified using an Ambion WT Expression kit (Affymetrix, Thermo Fisher Scientific, Waltham, MA, United States). Subsequently, cDNA was fragmented, labeled, and hybridized to the microarray using Affymetrix standard protocols. Affymetrix Porcine Snowball microarrays containing 47,880 probesets were used to determine expression profiles. Affymetrix Expression Console software was used for robust multichip average normalization and gene detection by applying detection above background algorithm. Expression data are available in the Gene Expression Omnibus public repository (GEO accession number GSE83932: GSM2221843-GSM2222139). Further filtering was done by excluding transcripts with low signals and probes that were present in <80% of samples. In total, 24,904 probes passed quality filtering and were used for further analyses. Both mRNA and SNPs were mapped to the porcine reference genome using Sscrofa 10.2 (Ensembl downloaded from NCBI^1^).

### Data Pre-processing and Statistical Analysis

After quality control and filtering for metabolites of low concentrations and samples with low concentrations of analytes as well as outlier animals, 74 out of 90 metabolites from 343 individuals were further analyzed. Z-score for each metabolite was calculated as: (relative metabolite level in the samples – mean of metabolite level in the samples)/*SD* of metabolite levels in the samples. Metabolite data were further pre-processed to account for systemic effects. Mixed-model analyses of variance using JMP Genomics (SAS Institute, Cary, NC, United States) were used to adjust for fixed and random effects. The genetic similarity matrix between individuals was first computed as identity-by-descent of each pair for the k-matrix and considered as a random effect. For control of population stratification, top principal components (PCs) explaining >1% of variation were considered as covariates. In total, 15 PCs were included as covariates. Gender was used as a fixed effect, batches of metabolite measurement were used as a random effect, and carcass weight was considered as a covariate. Residuals were retained for further analysis.

Metabolite QTL (mQTL) analyses were conducted using the R-package Matrix eQTL (Shabalin, 2012). Matrix eQTL tests for association between each SNP and residual metabolite levels by modeling the additive effects of genotypes in a least squares model (Shabalin, 2012). It performs a separate test for each metabolite–SNP pair and corrects for multiple comparisons by calculating the false discovery rate (FDR).

Residuals of mRNA transcript abundances, after correction for fixed effects (gender), random effects (genetic similarity matrix), and covariates (17 top PCs explaining >1% variation; carcass weight), were used to analyze eQTL by the same process used for mQTL in our previous study (Ponsuksili et al., 2016). We defined an eQTL as cis if an associated SNP was located within an area <1 Mb from the probeset/gene.

Residuals of mRNA and metabolite levels were used for pleiotropic association analyses to identify common regions. Multivariate analysis of variance (MANOVA) between residuals of metabolite and mRNA transcript levels and genetic marker data was used to analyze pleiotropic associations.

### Weighted Gene Co-expression Network Analysis (WGCNA)

Residuals of mRNA and metabolite levels were also used to construct co-expression/co-abundance networks using the blockwise modules function of the weighted gene co-expression network analysis (WGCNA) package in R (Langfelder and Horvath, 2008; Ponsuksili et al., 2015). Module–trait associations were estimated using the correlation between module eigengene which is the first PC of module of transcripts and of metabolites and plasma biomarkers. Correlations of metabolites with biochemical-clinical traits and mRNA transcript levels were estimated using Spearman coefficients and corrected for multiple comparisons by calculating FDR. Networks of genes and metabolites were visualized with Metscape 2^2^ (Karnovsky et al., 2012).

### NADP/NADPH Measurements

In order to validate the correlations found between transcripts and metabolites of the pentose phosphate pathway (PPP), NADPH concentration and NADP/NADPH ratio were measured from liver tissues of a random subset of animals (*n* = 27) using a NADP/NADPH assay kit (Abcam, Cambridge, United Kingdom) according to manufacturer’s instructions. Briefly, 50 mg of liver were washed and homogenized with extraction buffer and then centrifuged to isolate the NADPH/NADP+-containing supernatant. Supernatant was filtered through a 10-kD spin column to remove enzymes that may rapidly consume NADPH. An aliquot of supernatant was heated at 60°C for 30 min to decompose NADP+, cooled on ice, and spun quickly to remove the precipitate. Another aliquot of supernatant was not heated. Both aliquots were reacted with NADP+ cycling buffer and enzyme mix for 5 min at room temperature to convert NADP+ to NADPH. Solutions were then incubated with NADPH developer and absorbance was measured at 450 nm after 1, 2, or 3 h. Amount of NADPH (heated sample) and total NADP+ and NADPH (unheated sample) were quantified from a NADPH standard curve. In the same samples, expression levels of *HDAC4* and *G6PD*, which is the first limiting enzyme of PPP, were determined by qPCR validation. Three reference genes (*RPL32, RPS11*, and *ACTB*) were used, and all measurements were performed in duplicate.

### Cell Culture and siRNA Transfection

Human HepG2 cells were cultured in DMEM containing L-glutamine, 4.5 g/L D-glucose, and sodium pyruvate (Life Technologies) supplemented with 10% FBS, 100 U/mL penicillin, and 100 μg/mL streptomycin; the medium was refreshed every 2 days. Cell incubation was performed at 37°C in a humidified 5% CO_2_ atmosphere. Synthetic siRNAs were pre-designed by Qiagen. A total of four pre-designed siRNAs (Qiagen) per gene were first tested. The most two effective siRNA for *HDAC4* were used (Hs_HDAC4_3 FlexiTube siRNA and Hs_HDAC4_7 FlexiTube siRNA). The average values of negative non-silencing control siRNA (AllStars Negative Control siRNA, Qiagen), mock, and untreated were used as control. Transfection of siRNA was carried out using the HiPerFect transfection reagent (Qiagen) at 150 nM final concentration. The complexes were added drop-wise onto the cells, and the plates were then gently swirled to ensure uniform distribution of the transfection complexes. Forty-eight hours after siRNA transfection, cells were rinsed two times with PBS. The transfected cells were harvested for monitoring the effect of gene silencing. Three independent experiments were conducted. We determined the level of knockdown of *HDAC4* and *G6DP* using quantitative PCR (qPCR) (Roche, Germany) and normalized data using ß-actin as an internal control. All statistical analyses were performed using two-tailed Student’s *t*-tests.

## Results

**FIGURE 1:**
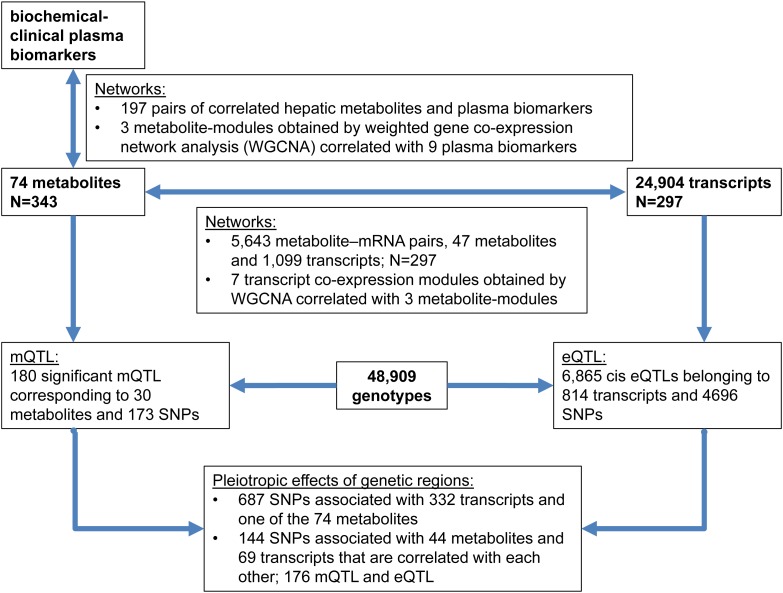
Outline of experimental flow and summary of main results.

The links between plasma biomarkers, hepatic metabolites, transcripts, and genotypes obtained from some 300 animals reared and performance tested under standardized conditions were analyzed and integrated in this study. Therefore, networks were obtained between metabolites and transcripts; both, from single and weighted correlation network analysis (WGCNA) of transcripts and metabolites (Langfelder and Horvath, 2008; Ponsuksili et al., 2015). Genetic regulation of metabolites (mQTL) was identified and integrated with a genome wide association study of transcripts levels (eQTL) (Ponsuksili et al., 2016). Pleiotropic effects of genetic regions that concertedly regulate transcripts and metabolites were considered. Finally, mQTL, eQTL, and phenotype of blood biochemical-clinical were integrated. The experimental flow is outlined in Figure 1.

### Metabolite Profiling

**FIGURE 2:**
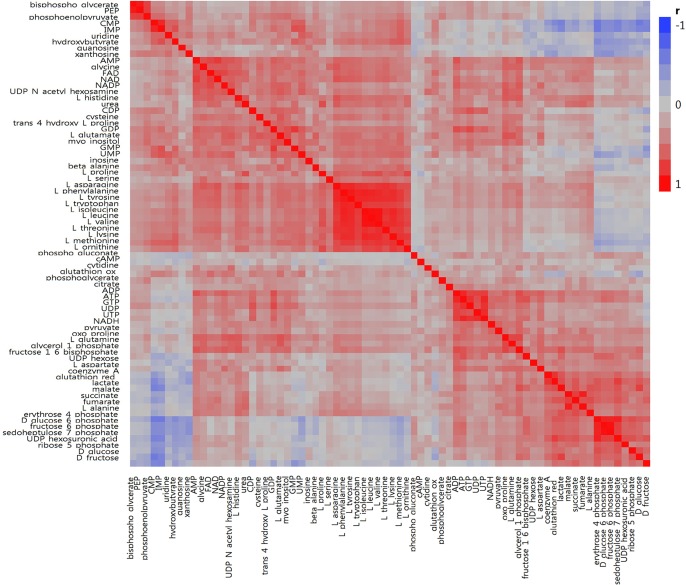
Correlation heatmap for 74 metabolites measured in porcine liver. Within the heatmap, red shows a positive correlation and blue shows a negative correlation.

**FIGURE 3:**
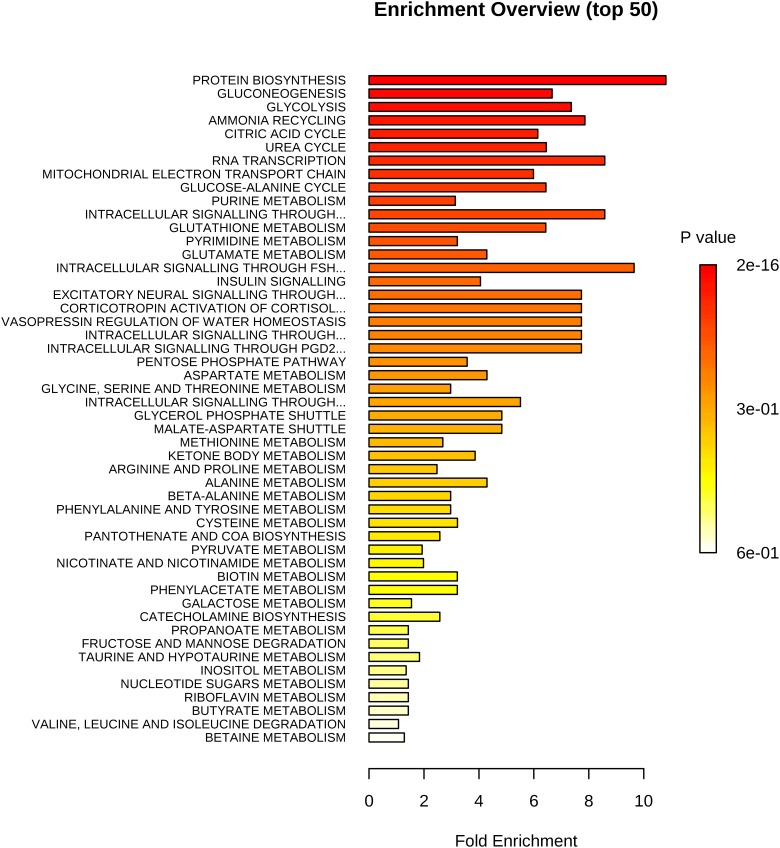
Enrichment analysis of 74 metabolites. Highest enrichment was found for protein biosynthesis (16/19) followed by gluconeogenesis (14/27) and glycolysis (12/21).

In total, we examined 74 liver metabolites of 343 pigs using mass spectrometry and found significant correlations between metabolites (Figure 2). Most metabolites in the same molecule class, such as amino acids or nucleotides, clustered together. Metabolite set enrichment analysis of 74 metabolites identified the highest enrichment for protein biosynthesis (16/19), followed by gluconeogenesis (14/27) and glycolysis (12/21) (Figure 3). Pathways which reached FDR < 5% are listed in Supplementary Table S1 together with metabolites within these pathways.

### Biochemical-Clinical Traits and Metabolites

**FIGURE 4:**
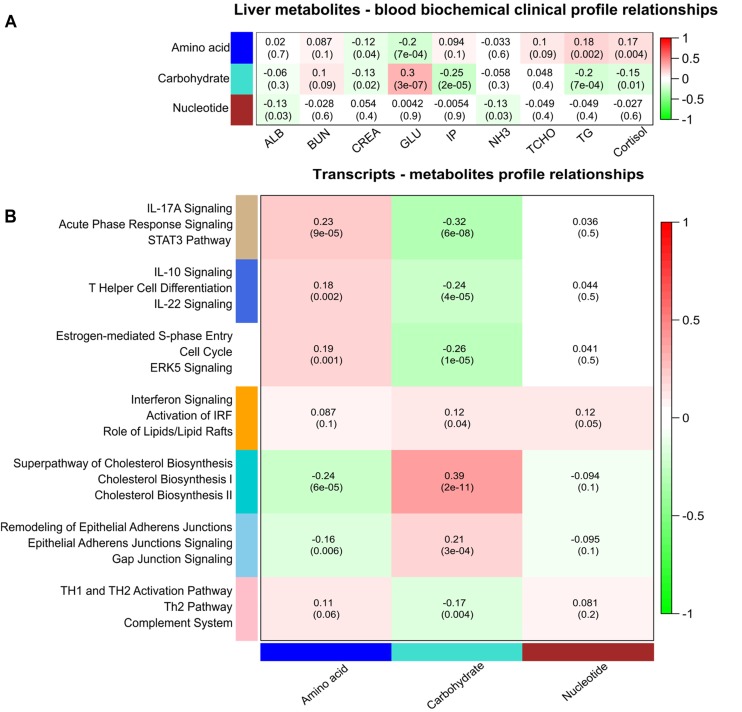
Correlation matrix of module eigengene values obtained for metabolites, transcripts, and plasma biomarkers. Weighted gene co-expression network analysis (WGCNA) groups metabolites and transcripts into modules based on correlated abundances. Each of the modules was labeled with a unique color as an identifier. **(A)** Three modules of metabolites including amino acids, carbohydrates, and nucleotides showing significant correlation with plasma biomarkers. **(B)** Seven modules of co-expressed transcripts showing significant correlation with three modules of metabolites. Within each cell, upper values are correlation coefficients and lower values are the corresponding *p*-values. Canonical pathways related to genes of these seven modules of co-expressed genes are given at the left side.

Liver metabolites were used for correlation analysis with approved plasma biochemical-clinical biomarkers (ALB; NH_3_; BUN; GLU; IP; CREA; and cortisol levels). Three main classes of metabolites with the same profile were identified using WGCNA including carbohydrates, amino acids, and nucleotides. Plasma GLU was found highly positively correlated with eigengene vector of the carbohydrate module and negative correlated with amino acid module (Figure 4A).

At a significance level of FDR < 5%, we identified 197 pairs of correlated hepatic metabolites and plasma biomarkers (Supplementary Table S2). Correlations between metabolites and biochemical-clinical traits ranged from 0.12 to 0.78. Overall, there was divergent correlation of biochemical-clinical biomarkers with carbohydrate- or amino acid-related metabolites on the one hand and nucleotide metabolism on the other hand. In particular, urea in liver was significantly correlated with BUN in plasma (*r* = 0.78; *p* < 10^-16^), as was liver D-glucose with plasma GLU (*r* = 0.45; *p* < 10^-16^). Significantly negative correlations were found between plasma GLU and cytidine monophosphate (CMP), inosine monophosphate (IMP), and guanosine monophosphate (GMP) (*r* = 0.56–0.29; *p* < 10^-8^). Plasma CREA was significantly negatively correlated with many amino acids, including L-isoleucine, L-tyrosine, L-leucine, L-threonine, L-valine, and L-asparagine (*r* = 0.13–0.17; *p* < 10^-3^). In addition, liver 4-hydroxyl-L-proline was significantly positively correlated with plasma CREA (*r* = 0.32; *p* = 1 × 10^-9^). Interestingly, plasma cortisol was significantly negatively correlated with liver D-glucose (*r* = 0.29; *p* = 1 × 10^-7^) and lactate (*r* = 0.28; *p* = 1 × 10^-7^) and positively correlated with IMP (*r* = 0.35; *p* = 9.9 × 10^-11^) and CMP (*r* = 0.30; *p* = 2.3 × 10^-8^).

### Transcripts and Metabolites

**FIGURE 5:**
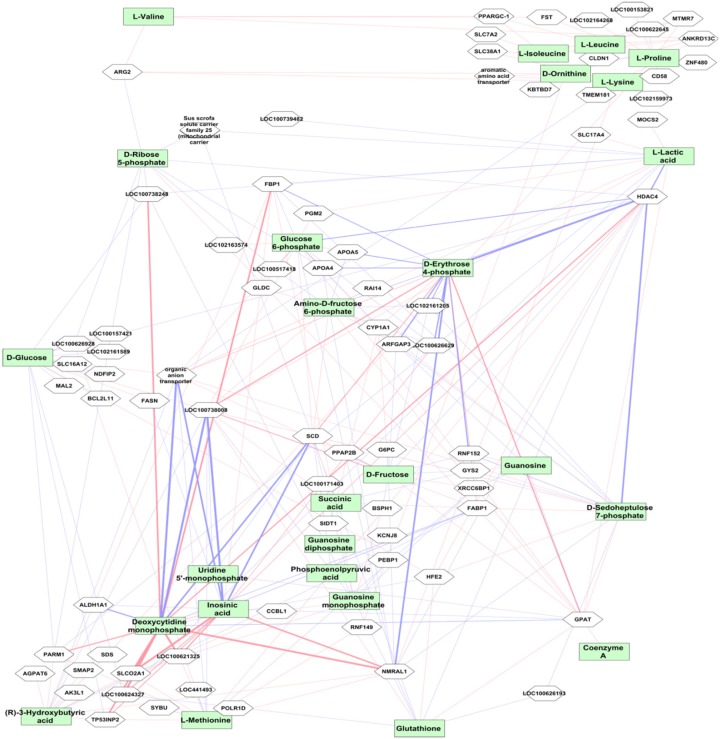
Correlation between mRNA transcript level and metabolites. The top metabolites with strong correlation to transcripts (*r* > ± 0.5, *p* < 10^-16^) are shown. The red connections indicate positive correlation and blue color shows negative correlation. The level of correlation is demonstrated by the thickness of the line. The metabolites are shown in green boxes and genes are in white boxes.

Weighted gene co-expression network analysis was performed using the transcriptome data from 24,904 liver transcripts. Seven modules of co-expressed transcripts were highly correlated with metabolite classes, as shown in Figure 4B. The co-expressed transcripts in each module were assigned to three top canonical pathways (Figure 4B). The amino acid module was significantly positively correlated with immune or acute phase response signaling, whereas the carbohydrate module was highly enriched in cholesterol biosynthesis. We explored transcriptional changes not only in terms of gene co-expression networks but also at the level of individual genes. Pair-wise correlations between the abundance of 24,904 liver transcripts and 74 metabolites in 297 individuals revealed 5643 metabolite–mRNA pairs with correlation coefficients of *r* > —0.40—, corresponding to *p* < 3.4 × 10^-12^ and FDR < 1.1 × 10^-9^. This covered 47 metabolites and 1099 annotated transcripts (1449 probesets). Supplementary Table S3 shows the top 20 transcripts that are correlated with the individual metabolites. A network-based approach was used to demonstrate the top relationship among transcripts and metabolites (Figure 5 and Supplementary Table S3). The most dominant pathways in these top pairs of metabolites and mRNA were related to PPP (D-ribose 5-phosphate, amino-D-fructose 6-phoshate, D-sedoheptulose 7-phosphate, D-erythrose 4-phosphate), purine (GMP, GDP, IMP), and pyrimidine metabolism (UMP and CMP).

Highly negative correlation was found between *LOC100738008* (thyroid hormone-inducible hepatic protein, *THRSP*) with IMP and CMP (*r* = -0.75 *p* < 10^-16^) followed by *HDAC4* with D-erythrose 4-phosphate (*r* = -0.69, *p* < 10^-16^). Expression levels of *HDAC4* were highly positively correlated with CMP, IMP, and UMP. In contrast, *HDAC4* levels were strongly negatively correlated with metabolites in carbohydrate metabolism, particularly PPP metabolites, including D-fructose, D-glucose, glucose 6-phosphate, D-erythrose 4-phosphate, fructose 6-phosphate, fumaric acid, L-lactic acid, malate, D-ribose 5-phosphate, D-sedoheptulose 7-phosphate, and succinic acid. In addition, strong positive correlation was found between CMP and *NMRAL1* (*r* = 0.72; *p* < 10^-16^). Furthermore, transcript levels of *ARG2*, followed by *SLC22A7* (organic anion transporter), *XRCC6BP1, SLC38A1*, and *SLC7A2*, were highly correlated with most amino acids.

### NADP/NADPH Measurements

Because PPP was dominantly linked with *HDAC4*, we measured NADPH concentration and the ratio of NADP/NADPH, i.e., the main products of PPP, as well as expression levels of *HDAC4* and *G6PD*, the key enzyme of PPP, in order to provide experimental evidence of the link of transcripts and PPP activity. Using qPCR, we found significant correlation between NADPH concentration and expression levels of *HDAC4* and *G6PD*. We confirmed expression levels of *HDAC4* obtained from the microarray by qPCR (*r* = 0.93; *p* < 0.0001) while *G6PD* was not available on the Affymetrix chip. Expression levels of *HDAC4* were positively correlated with NADP/NADPH (*r* = 0.78; *p* < 0.0001) and negatively correlated with NADPH concentration (*r* = -0.71; *p* < 0.0001). *G6PD* had a significant negative correlation with *HDAC4* (*r* = -0.44; *p* = 0.02) but positive correlation with NADPH concentration (*r* = 0.61 and *p* = 0.0007) and negative correlation with NADP/NADPH (*r* = -0.47; *p* = 0.012). *G6PD* expression also was correlated with PPP metabolites, including erythrose 4-phosphate, sedoheptulose 7-phosphate, D-glucose 6-phosphate, and fructose 6-phosphate (*r* = 0.58–0.63; *p* = 0.0012–0.0004).

### *HDAC4* Knockdown and *G6PD* Expression

**FIGURE 6:**
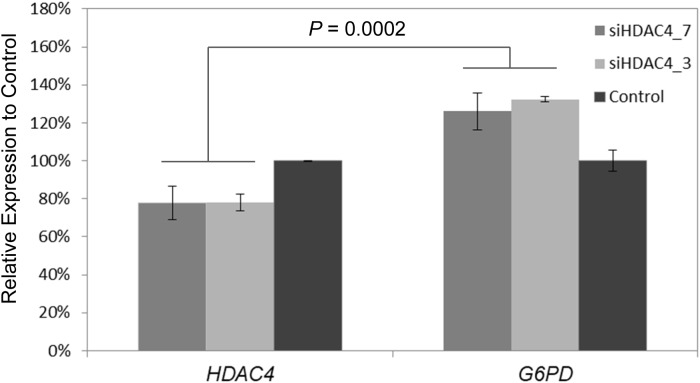
Knockdown of *HDAC4* by RNA interference reveals upregulation of *G6PD*. Two siRNAs siHDAC4_7 and siHADC4_3 were designed to target *HDAC4* and transfected into human HepG2 cells *in vitro*. Relative mRNA expression was measured by qPCR 48 h after transfection. Expression was normalized to ß-actin internal controls. Expression of *G6PD* was significantly increased relative to its expression in control cells and *HDAC4* levels at 48 h post-transfection of siRNA. The data represent means ± *SD* (*n* = 3).

To further experimentally elucidate the link of *HDAC4* and *G6PD* expression, RNAi was used to knockdown *HDAC4* expression *in vitro* in the Human HepG2 cells line. Subsequently, relative expression of *G6PD* was measured using qPCR. siRNA targeting *HDAC4* inhibited its expression to 70–80% relative to control cells (*p* < 0.004). At the same time, *G6PD* showed increased expression levels to 120–130% compare to control (*p* < 0.003) leading to pronounced differential expression between *HDAC4* and *G6PD* (*p* = 0.0002) (Figure 6).

### Genome-Wide Association of Metabolites (mQTL)

**FIGURE 7:**
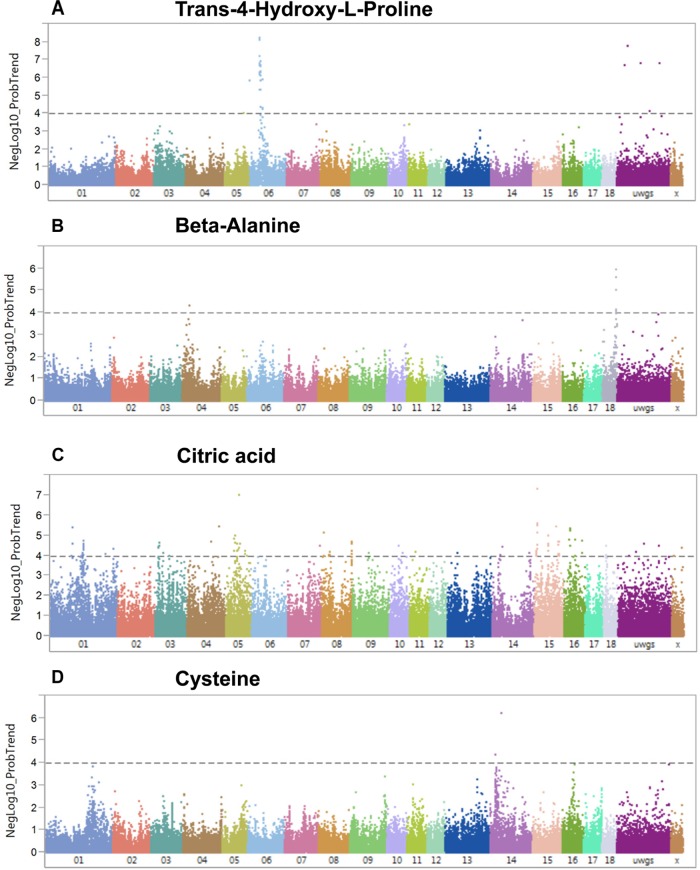
Manhattan plots visualizing genome wide associations of SNPs and metabolites (mQTL). **(A)** Trans-4-hydroxy-L-proline, **(B)** beta-alanine, **(C)** citric acid, and **(D)** cysteine. The dotted line depicts the genome-wide significance thresholds at negative log 10 > 4.

A genome-wide association study covering 48,909 SNP genotypes and 74 metabolites revealed 180 significant mQTL that corresponded to 30 metabolites and 173 SNPs at a threshold of –log_10_ > 4 (Supplementary Table S4). Table 1 lists top 10 associations. Only hydroxy-L-proline reached the significance threshold of FDR < 5% while other three metabolites (citrate, cysteine, and beta-alanine) showed suggestive mQTL at FDR ≤ 10%. Percent phenotypic variance explained by peak markers for these four metabolites was 6.7–9.4%. Figure 7 shows associations of these four metabolites across different pig chromosomes. The strongest association was for trans-4-hydroxy-L-proline with SNPs at 39.9 Mb on chromosome 6 (*p* = 6 × 10^-9^) (Table 1 and Figure 7A). Markers at position 53 Mb of chromosome 18 showed significant association with beta-alanine (Figure 7B). For citric acid (Figure 7C) and cysteine (Figure 7D), significant markers were mapped at various regions in the genome.

**Table 1:** Top 10 mQTL results.

Metabolite	SNP_ID	rs_number	*p*-value	SSC10_2	Base pair
Trans-4-hydroxy-L-proline	MARC0072609		6.04E-09	6	39960478
Citric acid	DRGA0014885		9.31E-08	15	11247606
Cysteine	ALGA0077013	rs80979261	6.46E-07	14	40660903
Beta_alanine	ASGA0085673	rs81327629	1.17E-06	18	53448342
Ornithine	ALGA0110895	rs81339246	2.50E-06	1	
Fumaric acid	ASGA0090623	rs81308979	5.20E-06	X	12973924
Malate	ASGA0090623	rs81308979	6.21E-06	X	12973924
L-Lactic acid	H3GA0017168	rs80890289	6.68E-06	5	99642466
6-Phospho-gluconate	CASI0009941	rs330835858	6.91E-06	16	63944017
NADH	ASGA0086564	rs81309680	7.96E-06	16	


### mQTL, eQTL, and Transcript Correlated Metabolites

Metabolic QTL regions contain numerous positional candidate genes, depending upon the level of linkage disequilibrium. To support and narrow down the number of candidate genes in regions, we integrated our previous eQTL data from the same pigs (Ponsuksili et al., 2016). Many SNPs associated with metabolites were also associated with transcripts. In our previous study, 6865 eQTLs were identified as cis, belonging to 1028 probesets (814 annotated transcripts) at FDR < 5% (*p* < 10^-7^). Further, 687 SNPs that were associated with mRNA transcripts (332 probesets) were associated with one of the 74 metabolites.

**FIGURE 8:**
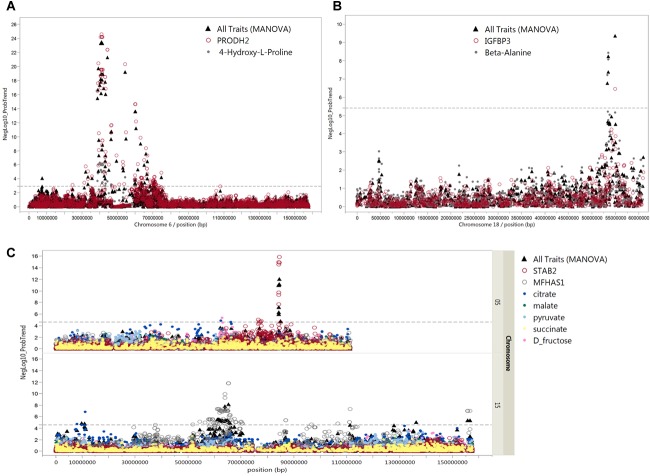
Pleiotropic associations of metabolites and mRNAs. Manhattan plots of pleiotropic associations between metabolites and mRNA expression. The pleiotropic association of these transcripts and metabolites (all traits) was significant (FDR ≤ 5%). **(A)** cis-eQTL of *PRODH2* and mQTL for trans-4-hydroxy-L-proline in close vicinity on chromosome 6, **(B)** cis-eQTL of *IGFBP3* and mQTL for beta-alanine in close vicinity on chromosome 18, and **(C)** cis-eQTL of *STAB2* on chromosome 5 and *MFHAS1* on chromosome 15 with mQTL for citrate, malate, pyruvate, succinate, and D-fructose. The *x*-axis indicates chromosome locations and *y*-axis shows –log10 of the *p*-values of multivariate analysis of variance (MANOVA). The dashed line shows the levels of *p*-values which are significant at 5% FDR.

In addition, we considered only metabolites that significantly correlated with mRNA transcripts at FDR < 5%. In total, 144 SNPs were associated with 44 metabolites and 69 metabolite-correlated transcripts, representing 176 mQTL and eQTL (Supplementary Table S5). Nineteen out of these 144 SNPs on *Sus scrofa* chromosome (SSC) 6 associated with trans-4-hydroxy-L-proline (*p* < 6.0 × 10^-9^–1.1 × 10^-4^). These SNPs were simultaneously associated with transcript levels of *PRODH2* (*p* < 4.7 × 10^-26^–4.9 × 10^-11^). Moreover, trans-4-hydroxy-L-proline was negatively correlated with *PRODH2* (*r* = -0.40; *p* = 1.6 × 10^-12^). Pleiotropic association analyses also showed SNP-directed links between trans-4-hydroxy-L-proline and *PRODH2* with 91 SNPs on SSC 6 (FDR < 5%) (Figure 8A).

At 5% FDR, six SNPs at position 53.4–54.9 Mb on SSC 18 were associated with beta-alanine and transcript levels of *IGFBP-3* (Figure 8B). The correlation between beta-alanine and transcript levels of *IGFBP-3* was *r* = –0.17 and *p* = 2.8 × 10^-3^. In other cases, SNPs located on SSC 7 position 20.5 Mb associated with transcript levels of *ALDH5A1* (*p* = 5.1 × 10^-13^) were also associated with beta-alanine, although at FDR > 5%. The correlation between *ALDH5A1* and beta-alanine was highly significant (*r* = –0.24; *p* = 2.7 × 10^-5^). The highest correlation was found between transcripts levels of *DPYS* and 3-hydroxybutyrate (*r* = –0.45; *p* = 2.6 × 10^-15^). Three SNPs located on SSC 4 position 35.6 Mb were associated with *DPYS* (*p* = 6.6 × 10^-11^) and, at a lower significance level, with 3-hydroxybutyrate (*p* = 1.9 × 10^-3^). As shown in Figure 7C, significant markers associated with citrate mapped to various regions in the genome. By combining eQTL, mQTL, and the correlation of corresponding mRNAs and metabolites, we found two interesting candidate genes in peak regions for citrate: *STAB2* on SSC 5 position 84.3 Mb and *MFHAS1* on SSC 15 position 63.7 Mb. Ten SNPs on SSC 15 position 63.7 Mb were associated with both *MFHAS1* (*p* = 8.2 × 10^-12^) and citrate (*p* = 3.4 × 10^-4^). Eight significant markers associated with *STAB2* (*p* = 1.1 × 10^-7^–1.1 × 10^-6^) were also associated not only with citrate but also with malate, succinate, pyruvate, and D-fructose (*p* = 8.9 × 10^-3^–4.4 × 10^-4^). These metabolites, which mostly belong to the citric acid cycle, were also negatively correlated with *STAB2* (*r* = 0.21–0.31; *p* = 2.4 × 10^-4^–5.3 × 10^-8^). Pleiotropic association analyses of transcript levels of both *STAB2* and *MFHAS1* and the metabolites of citrate, malate, succinate, pyruvate, and D-fructose showed 47 markers located on SSC 5, with 15 reaching a significance threshold of 5% FDR (Figure 8C). Another interesting transcript was *RBBP9*, which was negatively correlated with ribose 5-phosphate (*r* = 0.16; *p* = 4.5 × 10^-3^) and D-glucose 6-phosphate (*r* = 0.30; *p* = 2.9 × 10^-7^). Transcript levels of *RBBP9* were associated with 6 SNPs that were also associated with both ribose 5-phosphate and D-glucose 6-phosphate.

## Discussion

An improved understanding of non-genetic and genetic regulation of metabolite levels facilitates their interpretation as biomarkers for complex traits related to the metabolic status and in terms of exogenous and endogenous impacts on phenotypes. Moreover, identification of links between genetic polymorphisms and transcript and metabolite levels contributes to the elucidation of biomarkers that are the cause or consequence of changes in metabolic pathways. However, interpretation of mQTL data is demanding due to the fact that many metabolites are involved in various pathways. Here, we investigated a set of metabolites—mostly amino acids, carbohydrates, and nucleotides—in the polar phase of liver extracts.

### Correlation Between Biochemical-Clinical Traits, Transcripts, and Metabolites

To understand the relationship between gene expression, metabolite levels, and biochemical-clinical traits using a system genetics approach (Civelek and Lusis, 2014), we integrated these data obtained from the same pigs by calculating pair-wise correlations and WGCNA. We found significant intra- and inter-class correlations between metabolites especially amino acids and carbohydrate reflecting shared biochemical pathways or regulatory interactions with immune and cholesterol biosynthesis. The presence of significant correlations between metabolites categorized and biological function of co-expression transcripts presumably reflects either multiple roles of metabolites or interactions between metabolic pathways and immune system. Correlation of metabolites with transcripts can be due to enzymes, receptors, and signals of pathways encoded by corresponding genes or regulatory factors affecting gene expression. We identified many associations that show that the approach is suitable to identify biologically meaningful links between variation at the genome, transcriptome, and metabolome level with clinically relevant phenotypes. Thus, this approach has the potential to detect novel biomarkers while considering the contribution of exogenous and endogenous factors to individual variation.

For example, D-erythrose 4-phosphate, fructose 6-phosphate, D-ribose 5-phosphate, and D-sedoheptulose 7-phosphate, which belong to PPP, were highly negatively correlated with transcript levels of *HDAC4*. PPP is one of the fundamental components of cellular carbohydrate metabolism and is especially crucial for cancer cells (Kowalik et al., 2017). We confirmed the association by measuring ratio of NADP/NADPH and concentration of NADPH, for which PPP is the major source, as well as expression of *HDAC4* and *G6PD*. Here we show an association of PPP and *HDAC4* in healthy animals, indicating a possible epigenetic-based link between the histone-modifying *HDAC4* and the PPP-driving *G6PD*. *NMRAL1*, which encodes an NADPH sensor protein, is another transcript negatively correlated with PPP metabolites and contributes to regulation of the oxidative phase of PPP (Barcia-Vieitez and Ramos-Martínez, 2014). In addition, knockdown of *HDAC4* using RNAi was shown to be associated with increasing *G6PD* expression.

The liver plays a central role in processes of glycogenesis, glycogenolysis, and gluconeogenesis and thus glucose homeostasis (Nordlie et al., 1999). Our results demonstrate that plasma GLU is highly positively correlated with liver D-glucose. This also matches the finding that transcript levels of both *HDAC4* and *NMRAL1* are negatively correlated with plasma GLU and liver D-glucose, with the latter two being positively correlated.

Many transcripts positively correlated with plasma GLU and also correlated with liver metabolites like CMP and IMP, including *THRSP, SCD*, and *GPAM*, most of which are involved in lipid metabolism. Thyroid hormone responsive protein (THRSP) is involved in lipogenic processes and is associated with obesity (Ortega et al., 2010) and differential intramuscular fat in cattle (Hudson et al., 2015). Stearoyl-CoA desaturase (*SCD*) is a rate-limiting enzyme in fatty acid biosynthesis and thus a crucial control point of hepatic lipogenesis and lipid oxidation. Glycerol-3-phosphate acyltransferase (*GPAM*) encodes a mitochondrial enzyme that preferentially accepts saturated fatty acids as substrates for glycerolipid synthesis. Together, we show a link between liver metabolites and transcripts involved in lipid metabolism and plasma biochemical-clinical traits.

We found plasma cortisol levels were negatively correlated with liver metabolites that are mostly involved in glucose metabolism. Plasma cortisol levels also positively correlated with liver metabolites like CMP, IMP, and GMP, which in turn correlated with transcripts involved in lipid metabolism. This finding confirms our previous study, where we demonstrated these linked biological functions and molecular pathways using an integrative multi-omics approach (Ponsuksili et al., 2012).

Administration of two nucleotides, CMP and UMP, favors the entry of glucose in muscle and maintenance of hepatic glycogen levels during exercise (Gella et al., 2008). Interestingly, we found that cortisol-mediated homeostasis of lipid and carbohydrate metabolism in liver was associated with transcript levels of *CREM*. Abundance of *CREM* transcripts negatively correlated with plasma GLU and liver metabolites of carbohydrate metabolism (D-fructose, D-glucose, ribose 5-phosphate, erythrose 4-phosphate, sedoheptulose 7-phosphate, and lactate) and, at the same time, positively correlated with cortisol levels. *CREM* encodes a transcription factor that binds to cAMP responsive elements to mediate signal transduction during complex processes (Kirchhof et al., 2013; Ella et al., 2014). Previous studies show that *Crem* knock-out mice exhibit less anxious behaviors than wild-type mice (Maldonado et al., 1999). *CREM* is involved in cancer (Passon et al., 2012) and circadian regulation of cholesterol synthesis in the liver (Acimovic et al., 2008). Together, our results link hormone levels in plasma with metabolite and transcripts levels in liver.

*ARG2* encodes arginase, which is the enzyme of the final step of the ornithine-urea cycle converting L-arginine to L-ornithine and urea. In the present study, expression of *ARG2* was highly correlated with most amino acids, including L-isoleucine, L-leucine, L-lysine, L-methionine, L-ornithine, L-proline, and L-valine. These amino acids were also negatively correlated with plasma CREA. Transcript levels of *ARG2* also were negatively correlated with plasma CREA and positively correlated with plasma BUN. *Arg2*-/- mice have lower plasma CREA and BUN levels after renal injury (Raup-Konsavage et al., 2017). Our study shows that *ARG2* plays a central role for most amino acid metabolites in liver and is linked to biochemical properties of blood.

Our study highlights the value of integrating data from the same animals from various -omics levels, including transcriptome, metabolome, and biochemical-clinical traits that share biological pathways or functions. We found that epigenetic modifications mediated by *HDAC4* may play a significant role in PPP. Further, liver metabolites of the nucleotide class linked transcripts involved in lipid metabolism and cortisol. Finally, significant transcripts, such as *ARG2*, linked most amino acids in liver and biochemical-clinical traits, including CREA and BUN.

Comprehensive metabolite screens in the porcine model have identified novel associations among transcript levels, metabolites, and biochemical-clinical traits. Several studies have addressed the genetic regulation of metabolites serving as biomarkers for diseases (Illig et al., 2010; Wang et al., 2011; McMahon et al., 2017; Zhang et al., 2017). However, most studies have measured metabolites in blood serum or urine, while few have focused on genetic regulation of metabolites in other tissues, such as liver or fat (Ghazalpour et al., 2014; Parks et al., 2015). In this study, we integrated genetic-regulated liver metabolites, liver transcripts (mQTL and eQTL), and plasma biochemical-clinical traits. We prioritized genes based on cis-eQTL. For genome-wide significant loci associated with trans-4-hydroxy-L-proline, we identified *PRODH2* as significantly associated with the same SNPs. In addition, we demonstrated that these SNPs show pleiotropic effects by simultaneously affecting trans-4-hydroxy-L-proline and *PRODH2* expression. Further, we identified *PRODH2* as a high-confidence candidate gene within a locus associated with trans-4-hydroxy-L-proline, which in turn strongly correlated with plasma CREA. Trans-4-hydroxy-L-proline is metabolized by the liver and kidneys (Knight et al., 2009). Proline dehydrogenase 2 (*PRODH2*) catalyzes the first enzymatic step in the hydroxyproline catabolic pathway in liver and kidney mitochondria. In addition, *PRODH2* is reported as a molecular target for treating primary hyperoxaluria (Summitt et al., 2015). Mutations in *PRODH2* cause human hydroxyprolinemia, which hampers dehydrogenation of hydroxyproline to delta1-pyroline-3-hydroxy-5-carboxylic acid (Staufner et al., 2016).

In this study, we found a highly negative correlation between *DPYS* and 3-hydroxybutyric acid and identified three SNPs regulating both. Moreover, we found 3-hydroxybutyric acid correlated with cortisol. *DPYS* encodes dihydropyrimidinase, which is the second enzyme of the pyrimidine degradation pathway. The facts that patients with dihydropyrimidinase deficiency show mainly neurological and gastrointestinal abnormalities (van Kuilenburg et al., 2010) and that hydroxybutyric acid passes through the blood–brain barrier into the central nervous system (Sleiman et al., 2016) provide a possible link between *DPYS* and hydroxybutyric acid. Our study provides further evidence for this relationship. However, the link to cortisol as shown here is novel and still unclear.

IGF-binding protein-3 (*IGFBP-3*) is the major carrier protein for IGF-1 and plays a role in cancer, apoptosis, and pathogenesis of ischemia reperfusion after liver injury (Lee et al., 2014; Zhou et al., 2015; Wang et al., 2017). High *IGFBP-3* levels impact myogenesis and enhance muscle protein degradation (Huang et al., 2016). Patients with non-alcoholic steatohepatitis have increased levels of hepatic alanine (Kim et al., 2017). In this study, we found for the first time a link between genetic regulated alanine levels (mQTL) and *IGFBP-3* (cis-eQTL)x.

Genetically regulated metabolites belonging to the citrate cycle (D-fructose, malate, succinate, pyruvate, and citrate) share SNPs that also are associated with transcript levels of *STAB2* and *MFHAS1* (cis-eQTL). The biological function of both transcripts linked via common SNPs and to liver metabolites is still unknown. Here, SNPs located on SSC 17 position 27.4 Mb were associated with transcript levels of *RBBP9* (cis-eQTL) and also with ribose 5-phosphate and glucose 6-phosphate levels, both PPP metabolites. Glucokinase phosphorylates glucose to glucose 6-phosphate in liver as a substrate for several metabolic pathways, including PPP, which is particularly important in rapidly dividing cells like cancer cells for DNA replication. Further, previous studies have reported retinoblastoma binding protein 9 (*RBBP9*) is a tumor-associated protein in pancreatic neoplasia, affecting cell cycle control and contributing to the TGF-β signaling pathway (Acimovic et al., 2008; Vorobiev et al., 2012).

## Conclusion

In summary, this study is the first to combine metabolomics, transcriptomics, and genome-wide association studies in a porcine model. Our results improve understanding of the genetic regulation of metabolites which link to transcripts and finally biochemical-clinical parameters. Further, high-performance profiling of metabolites as intermediate phenotypes is a potentially powerful approach to uncover how genetic variation affects metabolic and health status. Our results advance knowledge in areas of biomedical and agricultural interest and identify potential correlates of biomarkers, SNPs-metabolites, SNPs-transcripts, and biochemical-clinical traits.

## Ethics Statement

Animal care and tissue collection procedures were approved by the Animal Care Committee of the Leibniz Institute for Farm Animal Biology and carried out in accordance with the approved guidelines for safeguarding good scientific practice at the institutions in the Leibniz Association and the measures were taken to minimize pain and discomfort and accord with the guidelines laid down by the European Communities Council Directive of 24 November 1986 (86/609/EEC).

## Author Contributions

SP and KW designed the study and interpreted the data. SP performed the statistical and bioinformatic analyses and drafted the manuscript. FH helped in bioinformatics analyses. EM and NT sampled the tissue probes and obtained biochemical-clinical data. KM and ML performed non-targeted metabolic profiling. FH, NT, EM, KM, ML, and KW critically revised the manuscript. All authors read and approved the final manuscript.

## Conflict of Interest Statement

The authors declare that the research was conducted in the absence of any commercial or financial relationships that could be construed as a potential conflict of interest.

## Footnotes

1 http://www.ncbi.nlm.nih.gov

2 http://cytoscape.org
