# Supplementary material for: Genetic Regulation of Liver Metabolites and Transcripts Linking to Biochemical-Clinical Parameters
Source: Front Genet. 2019 Apr 17;10:348. doi: 10.3389/fgene.2019.00348 (PMC6478805; doi:10.3389/fgene.2019.00348)
Supplement: Supplementary file 1 [file Data_Sheet_1.docx]

**Genetic regulation of liver metabolites and transcripts linking to biochemical-clinical parameters**

Siriluck Ponsuksili^1^, Nares Trakooljul^2^, Frieder Hadlich^1^, Karen Methling^3^, Michael Lalk^3^, Eduard Murani^2^, and Klaus Wimmers^2,4*^

^1^ Leibniz Institute for Farm Animal Biology (FBN), Institute for Genome Biology, Functional Genome Analysis Research Unit, Wilhelm-Stahl-Allee 2, D-18196 Dummerstorf, Germany

^2^ Leibniz Institute for Farm Animal Biology (FBN), Institute for Genome Biology, Genomics Research Unit, Wilhelm-Stahl-Allee 2, D-18196 Dummerstorf, Germany

^3^ Institute for Biochemistry – Metabolomics, University of Greifswald, Felix-Hausdorff-Str. 4, 17487, Greifswald, Germany

^4^ Faculty of Agricultural and Environmental Sciences, University Rostock, 18059 Rostock, Germany

*Corresponding author: wimmers@fbn-dummerstorf.de

**Supplementary Methods**

**GC-MS Setup and analysis**

GC-MS analysis was performed using an Agilent 6890N GC system equipped with an autosampler (model G2614A), an injector (model G2613A), and a coupled mass selective detector (model 5973N MSD) (Agilent). After injection of 2 µl of the sample the injection volume was split 1:25 at 250 °C, using helium as the carrier gas, with an inlet split flow of 10 ml/min and a pressure of 8.8 psi. Chromatographic separation was performed with a constant gas flow of 1 ml/min on a 30-m DB-5ms column (Agilent Technologies USA) with an 0.25 mm inner diameter and a 2.5 µm film thickness. The oven program started with an initial temperature hold at 70 °C for 2 min and continued with a heating rate of 10 K/min up to 150 °C, and 20 K/min up to 300 °C with a hold for 7 min. A transfer line at 280 °C was used to transfer the analytes to the mass selective detector and ionized by electron impact ionization at 230 °C. After a solvent delay of 6 min, mass spectra were acquired within a mass range of 50 to 500 atomic mass units, using a quadrupole temperature of 150 °C and scan acquisition mode.

Lyophilized samples were derivatized with 60 µl methoxyamine (20 mg/ml pyridine) for 90 min and afterwards with 120 µl *N*-methyl-*N-*trimethylsilyltrifluoroacetamide (Chromatographie-Service GmbH Germany) for 30 min both at 37 °C. Samples were centrifuged for 3 min at 25 °C and 13000 rpm and the supernatant was transferred to a new vial before injection. A sample containing 50 standard compounds was used as a daily quality control sample and injected each 5 samples within the batch.

Qualitative and quantitative analysis were performed using ChromaTOF software v4.50.8.0 (LECO Corporation USA). Metabolite identification was verified by matching the retention times and fragmentation patterns of detected peaks to those of analytical standard compounds measured within the same batch. A quantifier ion uniquely for each metabolite at the specific retention time was selected for quantification. The peak area of the quantifier ion of each metabolite was integrated and normalized to the area of the quantifier ion of the internal standard ribitol. This ratio represents the relative metabolite amount per liver sample (25 mg wet weight of liver per sample).

**HPLC-MS setup and analysis**

Lyophilized liver extracts and blank samples were dissolved in 100 µl water (HPLC-MS grade; VWR Germany). After centrifugation of the sample for 5 min at 4 °C and 13000 rpm an ion-pairing high-performance liquid chromatography-MS (HPLC-MS) analysis was performed by using an Agilent 1100 series liquid chromatographic system, consisting of a degasser, a quaternary pump, and a cooled autosampler coupled to a time of flight mass spectrometer (micrOTOF, Bruker Daltonik GmbH Germany). 5 µl of each sample were injected for chromatographic separation on a Synergi 2.5 µm Fusion RP column (2.0 mm x 50 mm, Phenomenex Germany) attached to a guard column of the same material. Mobile phases were A (95% H_2_O, 10 mM tributylamine, 15 mM acetic acid, 5% methanol, pH 5.0) and B (100% methanol). Using a flow rate of 0.3 ml/min and the HPLC system conditioned to 95% solvent A, the elution gradient started with 5 to 20% B in 1 min and continued with 20 to 45% B in 9.5 min, 45 to 60% B in 1 min, 60 to 100% B in 0.5 min, 100% B for 5 min, 0 to 100% A in 0.1 min, and 100% A for 6.9 min. MS analysis was performed using electrospray ionization and negative-ion polarity. Full scan mode was used over the mass range of 50 to 3000 *m/z*. MS calibration was done by using 16 different masses, ranging from 112.98 to 1,132.79 *m/z*, from a sodium formate solution (49.4% H_2_O, 49.4% isopropanol, 0.2% formic acid, and 10 mM NaOH) as the calibration tune mixture. The calibration tune mixture was injected at the beginning of each chromatographic run. The guard column was changed every 20 samples to avoid peak broadening and decrease in peak area of the metabolites. A mixture of 33 standard compounds was used as a daily quality control sample and injected each 5 samples within the batch.

Metabolite identification was verified by matching the retention times and *m/z* values of detected peaks to those of analytical standard compounds measured within the same batch using the software DataAnalysis v4.0 (Bruker Daltonik GmbH Germany). For 1,3-bisphospho-glycerate, identification was done by database alignment of the calculated accurate mass. The area of *m/z* of [M-H]^-^ of each metabolite was integrated and normalized to the integral of the area of *m/z* of [M-H]^-^ of the internal standard camphorsulfonic acid by using the software QuantAnalysis v2.0 (Bruker Daltonik GmbH Germany), resulting in the relative metabolite amount per 25 mg wet weight of liver sample. Masses detected in blank samples were excluded from the data analysis of biological samples.

74 metabolites measured by GC-MS and HPLC-MS, respectively according to the table

| **HPLC-MS** | **GC-MS** |
| --- | --- |
| ADP | beta-alanine |
| AMP | citrate |
| ATP | cysteine |
| bisphospho-glycerate | D-fructose |
| cAMP | D-glucose |
| CDP | D-glucose-6-phosphate |
| CMP | fructose-6-phosphate |
| coenzyme A | fumarate |
| cytidine | glycerol-1-phosphate |
| erythrose-4-phosphate | glycine |
| FAD | hydroxybutyrate |
| fructose-1,6-bisphosphate | lactate |
| GDP | L-alanine |
| glutathion (ox) | L-asparagine |
| glutathion (red) | L-aspartate |
| GMP | L-glutamate |
| GTP | L-glutamine |
| guanosine | L-histidine |
| IMP | L-isoleucine |
| inosine | L-leucine |
| NAD | L-lysine |
| NADH | L-methionine |
| NADP | L-ornithine |
| PEP | L-phenylalanine |
| phospho-gluconate | L-proline |
| ribose-5-phosphate | L-serine |
| sedoheptulose-7-phosphate | L-threonine |
| UDP | L-tryptophan |
| UDP-hexose | L-tyrosine |
| UDP-hexosuronic acid | L-valine |
| UDP-N-acetyl-hexosamine | malate |
| UMP | myo-inositol |
| uridine | oxo-proline |
| UTP | phosphoglycerate |
| xanthosine | phosphoglycerate |
|  | pyruvate |
|  | succinate |
|  | trans-4-hydroxy-L-proline |
|  | urea |
